# *N-3* fatty acids as preventive and therapeutic agents in attenuating PCOS complications

**DOI:** 10.17179/excli2019-1534

**Published:** 2019-07-25

**Authors:** Mina Salek, Cain C. T. Clark, Mohsen Taghizadeh, Sadegh Jafarnejad

**Affiliations:** 1 Research Center for Biochemistry and Nutrition in Metabolic Diseases, Kashan University of Medical Sciences, Kashan, I.R. Iran; 2 Centre for Sport, Exercise and Life Sciences, Coventry University, Coventry, United Kingdom

**Keywords:** n-3 PUFA, PCOS, infertility, metabolic disorder, obesity

## Abstract

To our knowledge, in spite of several trials exploring the beneficial effect of *n*-3 polyunsaturated fatty acids (PUFA) on polycystic ovary syndrome (PCOS), no comprehensive evidence has investigated the effects of *n*-3 PUFA consumption on PCOS complications. Therefore, our aim was to conduct a review to investigate the possible effect and related mechanisms. A comprehensive systematic search was conducted in Embase, MEDLINE/PubMed, Google Scholar, and SCOPUS, to identify studies investigating *n*-3 fatty acids as a preventative or therapeutic agent for the attenuation of PCOS complications. Subsequently, the impact of omega-3 on PCOS, omega-3 and inflammation, omega-3 and insulin resistance, omega-3 and adipokines, omega-3 and lipid metabolism, omega-3 and endothelial function and omega-3 and hormonal factors were discussed. There are multiple mechanisms by which *n*-3 PUFAs may exert their beneficial effects on PCOS, including anti-obesity, glycemic and hormonal hemostasis, anti-inflammatory, regulation of adipokine production and enhancement of endothelial function**.** *N*-3 PUFAs are a promising agent in relieving complications associated with PCOS. Although most of the studies in patients with PCOS reported an improvement in most complications after administration of omega-3 supplements, there is a distinct dearth of studies investigating the dietary intake of these types of fatty acids. Moreover, favorable effects regarding the improvement of dyslipidemia, regulation of adipokines, regulation of hormonal factors and enhancement of endothelial function are limited. Therefore, more trials are warranted to investigate palatable mechanisms for clarifying the metabolic and hormonal effects of these agents in PCOS.

## Introduction

Polycystic ovary syndrome (PCOS) is considered one of the most common female endocrine disorders with an approximate prevalence of 5-10 % (Rondanelli et al., 2014[98]; Dunaif, 1997[24]; Asuncion et al., 2000[5]), with sufferers at an increased risk of reproductive-related disorders (Rondanelli et al., 2014[98]; Goodarzi et al., 2011[36]). There are several characteristics manifest in this syndrome including polycystic ovaries, irregular menstruation, hyperandrogenism and obesity and its associated consequences, such as insulin resistance, which is reported in about 5 to 20 percent of women, depending upon the applied diagnostic criteria (Faghfoori et al., 2017[28]; Pourghassem Gargari et al., 2015[89]). According to the Rotterdam ESHRE/ASRM sponsored PCOS consensus workshop, a positive diagnosis is considered when any two of the following three features are observed; oligo-ovulation/anovulation, polycystic ovaries on ultrasound examination and/or clinical and/or biochemical signs of hyperandrogenism (Rondanelli et al., 2014[98]; Rotterdam ESHRE/ASRM-sponsored PCOS consensus workshop group, 2004[99]). Additionally, there are several endocrine abnormalities in PCOS which result in metabolic alterations, such as; insulin resistance (IR), hyperinsulinemia, central obesity and metabolic syndrome (MeTS) (Rondanelli et al., 2014[98]), and their associated consequences, including increased risk of hypertension, developing type 2 diabetes (T2DM), dyslipidemia, cardiovascular disorders and atherosclerosis (Rondanelli et al., 2014[98]; Solomon, 1999[104]). The reasons for the manifestation of PCOS is not well established; despite this, a combination of parameters, including genetic and/or epigenetic, environmental factors and exposure to high levels of androgen prenatally are considered to, putatively, play a role in the onset of the disorder. Previous studies have revealed environmental factors may represent fundamental factors in the incidence and treatment of the disorder. Among these environmental factors, dietary patterns are considered as one of the most important and controllable factors (Faghfoori et al., 2017[28]; Nardo et al., 2008[78]; Dumesic et al., 2007[23]; Xita and Tsatsoulis, 2006[115]).

Although optimal treatment for PCOS is not yet fully determined; multifactorial approaches are advisable, including a combination of one or more of the most effective interventional strategies, such as diet and lifestyle changes, or the use of pharmaceuticals (hormonal agents: oral contraceptives/cyclic progestins, gonadotropins, antiestrogens, and metabolic agents such as insulin sensitizers). It has been shown in a recent survey of 138 endocrinologists and 172 gynecologists, performed by Cussons et al. (2005[16]) and Liepa et al. (2008[66]), that the majority of experts believe diet and exercise is the first step in the treatment of PCOS (Liepa et al., 2008[66]). For instance, dietary fat, such as essential poly-unsaturated fatty acids (PUFA) including omega-3 and omega-6 fatty acid, has been studied in relation to PCOS (Liepa et al., 2008[66]). Dietary sources of *n*-3 PUFA are limited in comparison with *n-*6 PUFA sources. Αlpha-linolenic acid (ALA) is synthesized by plants and is abundant in sources such as flaxseed and nuts or leaf (Geetha and Chakravarthula, 2018[32]), whilst fish are considered among the richest sources of *n*-3 PUFA eicosapentaenoic acid (EPA) and docosahexaenoic acid (DHA) (Albracht-Schulte et al., 2018[2]; Ratnayake and Galli, 2009[94]; Jafarnejad and Sadegh, 2011[47]; Li et al., 2018[65]). Omega-3 PUFAs are asserted to exert their favorable effects via the modulation of the amount and type of eicosanoids and by the regulation of both intracellular signaling pathways and transcription factor activities (Gray et al., 2013[38]; Serhan et al., 2004[101]; Babcock et al., 2003[6]). Gene expression is another example of the possible mechanism of *n*-3 PUFA consumption in positively affecting health indices (Gray et al., 2013[38]; Ren et al., 1997[95]). It seems that each of the aforementioned mechanisms, or indeed a combination of them, contributes to the regulation of different aspects of PCOS, including inflammation, blood pressure regulation, platelet adhesion and heart rhythm (Gray et al., 2013[38]; Harris et al., 2008[42]).

It has been shown in previous trials that consumption of unsaturated fat-rich foods may decrease the risk of chronic diseases; which is particularly true regarding risk factors for metabolic disorders seen in PCOS patients, including dyslipidemia, impaired endothelial function, and insulin resistance (Faghfoori et al., 2017[28]; Zivkovic et al., 2007[119]). To our knowledge, in spite of several trials exploring the beneficial effects of *n*-3 PUFA on PCOS (Table 1(Tab. 1); References in Table 1: Amini, 2018[3]; Cussons, 2009[17]; Ebrahimi, 2017[25]; Jamilian, 2018[48]; Kalgaonkar, 2011[51]; Khani, 2017[56]; McEwen, 2017[69]; Mejia-Montilla, 2018[70]; Mirmasoumi, 2018[73]; Mohammadi, 2012[74]; Nadjarzadeh, 2015[77]; Nomura, 2018[80]; Oner, 2013[83]; Phelan, 2011[87]; Rafraf, 2012[92]; Rahmani, 2017[93]; Talari, 2018[107]), no comprehensive review regarding the effects of *n*-3 PUFA consumption on PCOS complications has taken place. Therefore, our aim was to conduct a review to investigate the possible effect and related mechanisms of *n*-3 PUFA consumption on PCOS complications.

## Impact of Omega-3 on PCOS Complications:

### Omega-3 and obesity

Overweight and obesity are observed in 40 %-50 % of patients with PCOS and is considered a major risk factor for PCOS (Liepa et al., 2008[66]; Carmina and Lobo, 1999[13]). The accumulation of excess weight can lead to adipose tissue dysfunction, a phenomenon that is mechanistically connected to the pathogenesis of MetS and complications such as insulin resistance in liver and skeletal muscle (Albracht-Schulte et al., 2018[2]; Kim and Moustaid-Moussa, 2000[58]). It is assumed that lower serum *n*-3 PUFA concentrations (Albracht-Schulte et al., 2018[2]; Karlsson et al., 2006[55]), particularly DHA (increase of *n-6:n-3* ratio) (Albracht-Schulte et al., 2018[2]; Karlsson et al., 2006[55]), are associated with obesity, elevated waist circumference (Albracht-Schulte et al., 2018[2]; Micallef et al., 2009[71]) in adolescents (Karlsson et al., 2006[55]; Albracht-Schulte et al., 2018[2]), and adults with obesity (Albracht-Schulte et al., 2018[2]; Micallef et al., 2009[71]). Adipose tissue is considered an active endocrine organ which has the ability to secrete several adipokines and hormones, such as leptin and adiponectin, and cytokines, such as interleukin-6 (IL-6) (Albracht-Schulte et al., 2018[2]; Kalupahana et al., 2012[52]).

One of the primary treatments in managing obesity and its comorbidities is lifestyle modification with the aim of significant weight loss (Albracht-Schulte et al., 2018[2]). Natural bioactive compounds, such as *n*-3 PUFA, possess few side effects and could be considered a safe approach in comparison to other modalities of treatment (Albracht-Schulte et al., 2018[2]). There are a variety of putative mechanisms by which *n*-3 PUFA, particularly EPA and DHA, could work in improving body composition, modulating energy metabolism and reducing body weight (Albracht-Schulte et al., 2018[2]).

Adipocyte differentiation, which is considered a complex process in making adipose tissue, is mainly regulated by two different families of transcription factors, including peroxisome proliferator-activated receptor γ (PPAR-γ) and CCAAT/enhancer-binding proteins (C/EBPs) (Martinez-Fernandez et al., 2015[68]). It has been shown that DHA, in dosages of ≥ 50 μM, could facilitate the differentiation of adipocytes by up-regulation of mRNA levels of adipocyte protein 2 (aP2) and C/EBPα, PPARγ in 3T3-L1 cell lines (Martinez-Fernandez et al., 2015[68]; Murali et al., 2014[76]). Furthermore, accumulation of lipid droplets results in increased levels of PPARγ following administration of EPA (250 μM) (Martinez-Fernandez et al., 2015[68]; Hanada et al., 2011[40]). Additionally, previous studies have highlighted that *n*-3 PUFAs can modulate the apoptosis of adipocytes (Martinez-Fernandez et al., 2015[68]; Kim et al., 2006[57]). Collectively, it seems that marine-origin *n*-3 PUFAs may have the potential to regulate the adipocyte number and size via modulating adipocyte differentiation and apoptosis (Martinez-Fernandez et al., 2015[68]). Indeed, several studies have reported favorable outcomes of omega-3 consumption on obesity. For instance, in Oner and Muderris (2013[83]), BMI decreased significantly with daily oral administration of 1,500 mg omega-3 for 6 months (Oner and Muderris, 2013[83]). In another trial, which was conducted in PCOS patients, the subjects consumed omega-3 in doses of 2 g/day for 6 months, after which, waist circumference (WC) was significantly lower in those that consumed omega-3 vs. the control group (Khani et al., 2017[56]). Similar outcomes were reported in McEwen (2017[69]), where participants received 2 capsules of omega-3 PUFA acids per day, and at the end of the trial, WC was significantly lower in the omega-3 group vs. the control group (McEwen, 2017[69]). The findings of combination therapy have also shown promising effects, for instance, BMI decreased significantly following administration of pitavastatin and, either, EPA 1,800 mg or sarpogrelate 300 mg for 12 months in diabetic patients with arteriosclerosis (Nomura et al., 2018[80]). Conversely, it has been reported that consuming 4 g of omega-3 PUFA capsules, per day, for 8 weeks did not elicit any significant effect on weight, BMI, waist circumference, or waist to hip ratio (Rafraf et al., 2012[92]). However, the contradictory results are limited, and most previous studies have reported favorable effects of *n*-3 PUFA supplementation on obesity.

### Omega-3 and insulin resistance

Insulin resistance is defined as a condition in which the response of peripheral tissue to insulin is reduced (Lepretti et al., 2018[63]). This disorder occurs mostly in the skeletal muscle and the liver, with several negative impacts on glucose metabolism, which is mostly attributed to the defective regulation of glucose transporter isoform 4 (GLUT4). It seems that the skeletal muscle may play a substantial role in insulin resistance (Lepretti et al., 2018[63]; DeFronzo and Tripathy, 2009[19]; Lark et al., 2012[61]). Impaired suppression of gluconeogenesis, glycogenolysis in the liver, and suppression of glucose output are among the complications associated with insulin resistance (Home and Pacini, 2008[44]; Boden, 2011[8]; Lepretti et al., 2018[63]). The impaired suppression of lipolysis observed in white adipose tissue (WAT) contributes to hyperlipidemia in insulin-resistant subjects (Lepretti et al., 2018[63]; Boden, 2011[8]). Brown adipose tissue (BAT) also plays an essential role in glucose metabolism (Lepretti et al., 2018[63]; Stanford et al., 2013[105]), where it is demonstrated that the whole-body energy homeostasis can be regulated by BAT, with the dissipation of chemical energy as heat via mitochondrial uncoupling protein 1 (UCP1).

Insulin resistance has been reported in 30 % of lean and 75 % of obese patients with PCOS, whilst the severity of insulin resistance is more than would be expected for a given age and body weight (O'Connor et al., 2010[81]; Jonard and Dewailly, 2004[49]). The observed fasting hyperinsulinemia in obese PCOS patients is secondary to increased basal insulin secretion rates (Dunaif, 1997[24]; O'Meara et al., 1993[82]). Obesity results in insulin resistance, which is accountable for the pathogenesis of MetS (Albracht-Schulte et al., 2018[2]; Kim and Moustaid-Moussa, 2000[58]). Previous studies have demonstrated an improvement in insulin resistance after weight loss interventions) (Albracht-Schulte et al., 2018[2]; Kim and Moustaid-Moussa, 2000[58]) and several studies recommend that insulin resistance leads to significant disturbances in both metabolic and reproductive homeostasis, and thus may possess an essential role in the pathogenesis of PCOS (Gunalan et al., 2018[39]; Diamanti-Kandarakis and Dunaif, 2012[21]). In brief, insulin may exert its role in hyperandrogenism in PCOS pathophysiology via two distinct pathways: 1) stimulation of androgen production of theca cells with luteinizing hormone (LH) and increased androgen production which results in hirsutism and infertility. 2) Suppression of sex hormone-binding globulin (SHBG) synthesis in the liver (Gunalan et al., 2018[39]; Ehrmann, 2005[26]). SHBG is considered to be a plasma protein for androgen and estrogens; therefore, any decrement in SHBG levels could result in hyperandrogenism in PCOS sufferers. Metabolically, insulin plays an integral role in regulating glucose homeostasis, the activation of amino acid transportation, and suppression of lipolysis (Gunalan et al., 2018[39]; Kahn, 1994[50]). Mechanistically, it has been suggested that omega-3 PUFA may have beneficial effects on insulin sensitivity by attenuating endoplasmic reticulum (ER) stress, and increasing β-oxidation of mitochondrial fatty acid and mitochondrial uncoupling, with a concomitant reduction in lipid deposits and reactive oxygen species (ROS) production. Consequently, inflammatory processes are down-regulated, thereby enhancing insulin sensitivity. Another putative mechanism is related to mitochondrial fusion proteins - mitofusins 2 (Mfn2), which is involved in the maintenance of mitochondrial dynamics homeostasis and integrity of mitochondria-associated endoplasmic reticulum membrane (MAM). In the case of cellular stress, MAM integrity and mitochondrial fusion phenotype may facilitate maintenance of insulin sensitivity (Lepretti et al., 2018[63]).

There are some studies that show the effect of omega-3 supplementation on insulin resistance. In the study of Oner and Muderris (2013[83]) insulin levels significantly decreased following 6 months of omega-3 consumption; whereas there were no reported significant changes in glucose levels and HOMA index. In another trial, 30 participants consumed 2 g/day fish oil omega-3 fatty acid for 12 weeks; following which, a significant increase in the quantitative insulin sensitivity check index, serum insulin levels, and HOMA-IR were reported, respectively (Amini et al., 2018[3]). Mirmasoumi et al. reported a decrease in insulin values and homeostasis model of assessment-estimated insulin resistance, and an increase in quantitative insulin sensitivity check index after administrating 1 g/day flaxseed oil omega-3 fatty acids for 12 weeks (Mirmasoumi et al., 2018[73]). Similarly, a significant decrease in insulin, homeostasis model of assessment-estimated insulin resistance and a significant increase in quantitative insulin sensitivity check index were observed after 12 weeks intervention of omega-3 fatty acids and vitamin E co-supplementation (Ebrahimi et al., 2017[25]). However, in a recent meta-analysis which investigated the effect of omega-3 PUFA supplementation on insulin resistance in women with PCOS, no beneficial effect on insulin resistance and HOMA−IR was observed in comparison to the placebo group (Sadeghi et al., 2017[100]). 

### Omega-3 and inflammation

Chronic low-grade inflammation is normally involved in the pathogenesis of obesity-related disorders. Leukocytes in the adipose-tissue and circulation can enhance the insulin resistance in type 2 diabetes and obesity (Escobar-Morreale et al., 2011[27]; Fernandez-Real and Ricart, 2003[30]). Polycystic ovary syndrome contributes to a pro-inflammatory state (Escobar-Morreale et al., 2011[27]), and an association between molecular level inflammation and insulin resistance has been well reported (Escobar-Morreale et al., 2011[27]; González et al., 2006[34][35]). Moreover, there are elevations in several circulating pro-atherogenic inflammatory modulators in PCOS (Escobar-Morreale et al., 2011[27]; Diamanti-Kandarakis et al., 2006[22][20]; Lewandowski et al., 2006[64]; Hu et al., 2006[46]), which are accompanied by glucose-stimulated up-regulation of pro-atherogenic molecular pathways (Escobar-Morreale et al., 2011[27]; González et al., 2006[34]). However, it remains unknown whether the pro-inflammatory state and increase of circulating inflammatory mediators in PCOS are related to PCOS itself, or are primary manifestations of the inflamed status of adipose tissue (Escobar-Morreale et al., 2011[27]; Carmina et al., 2007[12]).

*N*-3 PUFAs influence the production of several inflammatory markers, such as adhesion molecules and cytokines (Calder, 2017[11]), whilst the most focused markers are the classic pro-inflammatory cytokines, tumor necrosis factor (TNF)-α, IL-6 and interleukin (IL)-1β. Many cell culture and animal reports, respectively, have reported that EPA/DHA can reduce the formation of TNF-α, IL-6, and IL-1β in response to lipopolysaccharide (LPS) exposure (Calder, 2017[11], 2015[9]). Whilst other studies have shown that EPA and DHA may lead to an increase in the cytokine IL-10 concentration, which is anti-inflammatory (Calder, 2017[11], 2015[9]). Additionally, it has been reported that *n*-3 PUFAs, especially DHA, could decrease the expression of adhesion molecules, such as intercellular adhesion molecule (ICAM)-1 and vascular cell adhesion molecule (VCAM)-1, on the surface of monocytes and endothelial cells (Calder, 2017[11], 2015[9]). It seems that the alterations in the expression of genes responsible for encoding the cytokines and inflammatory mediators induce the anti-inflammatory effects of *n*-3 PUFAs (Calder, 2017[11], 2015[9]). This suggests that *n*-3 PUFAs impact the signaling pathways, and, as a consequence, control gene expression in inflammatory cells.

Another mechanism by which EPA and DHA might have an effect on nuclear factor kappa B (NF-κB) activation, and the ability to induce transcription of pro-inflammatory genes, is related to PPAR-γ. This is considered to be a transcription factor which acts in an anti-inflammatory manner, which is likely attributable to its interference with the translocation of NF-κB to the nucleus. It has been reported that *n*-3 PUFAs can induce the PPAR-γ (Calder, 2017[11]; Forman et al., 1997[31]; Krey et al., 1997[60]) and DHA, specifically, can activate PPAR-γ in dendritic cells (Calder, 2017[11]; Kong et al., 2010[59]); which is accountable for inhibition of NF-κB activation and reduced production of the pro-inflammatory cytokines, including TNF-α and IL-6 and LPS stimulation (Calder, 2017[11]; Kong et al., 2010[59]). Furthermore, PPAR-γ target genes in dendritic cells are induced by DHA (Calder, 2017[11]; Zapata-Gonzalez et al., 2008[117]), suggesting that it may be a considerable anti-inflammatory mechanism attributable to DHA. Similarly, EPA derivatives, 15-deoxy-PGD3 and PGD3, can induce PPAR-γ in adipocytes, which results in the activation of the anti-inflammatory adipokine adiponectin production (Calder, 2017[11]; Lefils-Lacourtablaise et al., 2013[62]).

There are several reports suggesting that *n*-3 PUFA can improve inflammatory status in PCOS patients (Jamilian et al., 2018[48]; Talari et al., 2018[107]). In one study, co-supplementation of 3 g/day omega-3 from fish oil, with 50000 IU vitamin D, significantly decreased serum high-sensitivity C-reactive protein (hs-CRP), and down-regulated gene expression of IL-1; however, there was no significant effect on gene expression of IL-8, TNF-α or transforming growth factor beta (TGF-β) (Jamilian et al., 2018[48]). In another study, flaxseed oil omega-3 fatty acid supplementation resulted in a significant decrease in hs-CRP (Mirmasoumi et al., 2018[73]), whilst Rahmani et (2017[93]) demonstrated that consumption of 1 g/day omega-3 fatty acids from fish oil, for 12-weeks, resulted in up-regulation of PPAR-γ and down-regulation in gene expression of IL-1 and interleukin-8 (IL-8) (Rahmani et al., 2017[93]). Evidently, most previous studies emphasize the efficacy of *n*-3 PUFA administration in relieving inflammatory status in PCOS patients. 

### Omega-3 and adipokines

The adipokine alteration is considered to be a specific complication of PCOS, yet it is not fully understood whether such dysregulation is secondary to PCOS complications such as obesity, hyperinsulinemia, and hyperandrogenism, or directly related to PCOS (Baldani et al., 2019[7]). Adiponectin and leptin are among the adipokines secreted from adipocytes (Farimani et al., 2018[29]; Das et al., 2013[18]), and it has been shown that different adipokines can promote both inflammatory and anti-inflammatory activities (Farimani et al., 2018[29]; Nomura et al., 2009[79]). It seems that adiponectin expression as an anti-inflammatory adipokine is reduced in obese individuals, whilst expression of leptin as an inflammatory adipokine is elevated, compared to the lean subjects (Farimani et al., 2018[29]; Kang et al., 2016[53]; Ouchi et al., 2011[84]). The higher leptin concentration in obese subjects, which is due to leptin resistance, is related to higher concentrations of inflammatory markers, including TNF-α and CRP. In addition to the inflammatory responses of adipokines, it has been suggested that adiponectin and leptin can regulate appetite and energy expenditure, which results in an alteration of insulin sensitivity. (Farimani et al., 2018[29]; Gray et al., 2013[38]). *N*-3 PUFA's derived from plant and marine sources have been introduced as potential factors affecting the concentration of inflammatory and non-inflammatory adipokines (Farimani et al., 2018[29]; von Frankenberg et al., 2014[111]). Elevation of circulating adiponectin levels and reduced levels of leptin have been reported after *n*-3 PUFAs administration in previous studies (Farimani et al., 2018[29]; von Frankenberg et al., 2014[111]; Hariri et al., 2015[41]), which is associated with improved function of skeletal muscle mitochondria, and, consequently, increased glucose uptake. *N*-3 PUFA normalizes the secretory activity of adipocytes and improves adiponectin-mediated insulin sensitivity in skeletal muscle and the liver (Farimani et al., 2018[29]). Moreover, this can stimulate the activation of 5′AMP-activated protein kinase (AMPK) and PPARγ, which regulate metabolism by increasing glucose consumption and fatty acid oxidation in the liver and skeletal muscle (Farimani et al., 2018[29]; Gray et al., 2013[38]; Perez-Matute et al., 2007[86]; Tomas et al., 2002[109]; Yamauchi et al., 2003[116]). However, it seems that the effect omega-3 elicits on adipokine levels is equivocal, and widely dependent on several parameters, such as diet composition, the physiological and metabolic status of subjects (Farimani et al., 2018[29]; Moreno-Aliaga et al., 2010[75]). 

There are several studies related to the effect of omega-3 fatty acids on adipokine secretion, for instance, Mohammadi et al. reported an increase of adiponectin by 19.5 % via inducing omega-3 fatty acids supplementation for 8 weeks (Mohammadi et al., 2012[74]). In another study by Mejia-Montilla et al. (2018[70]), PCOS patients treated with omega-3 fatty acids for 12 weeks significantly increased mean levels of adiponectin. Similarly, in a study conducted in PCOS sufferers, following omega-3 supplementation (3 caps/ day, each containing 180 mg EPA and 120 mg DHA) for 8 weeks, mean adiponectin concentration increased in the omega-3 group, whilst visfatin concentration remained unchanged (Nadjarzadeh et al., 2015[77]). In Rafraf et al. (2012[92]), patients with PCOS took 4 g omega-3 fatty acids capsules per day, providing 1200 mg *n-3* long-chain polyunsaturated fatty acids (*n*-3 LC PUFA), for 8 weeks. The incumbent changes in serum visfatin levels were not significant in either the intervention or placebo groups (Rafraf et al., 2012[92]). In summary, the studies investigating the effects of *n*-3 PUFA on adipokines, particularly in PCOS, are limited, and further studies are warranted to clarify the possible efficacy of, and mechanisms associated with, improving PCOS complications. 

### Omega-3 and lipid metabolism

Significant changes in the production of FFAs, phospholipids, and bioactive lipids have been observed in obese and lean women with PCOS. The reported dyslipidemia in the PCOS population is characterized by reduced high density lipoprotein (HDL) cholesterol concentrations as the main outcome, and possible elevated levels of triacylglycerol (TAG), very low density lipoprotein (VLDL) and/or atherogenic non-A low density lipoprotein (LDL) cholesterol pattern as the secondary outcome (O'Connor et al., 2010[81]; Westerveld et al., 2008[113]). 

There are several studies that have reported an inverse association between plasma HDL-C levels and the incidence of CVD disorders (Pizzini et al., 2017[88]; Rader, 2003[91]; Kannel et al., 1964[54]; Assmann et al., 2002[4]), which emphasizes the potential atheroprotective effects of higher concentrations of HDL-C (Pizzini et al., 2017[88]; Siddiqi et al., 2015[103]). The beneficial effect of HDL as a CVD protecting factor is mediated by the Reverse Cholesterol Transport (RCT) activity of HDL-C. This process explains the cholesterol clearing pathways of peripheral, subendothelial macrophage, and fibroblast-derived cholesterol via direct (via hepatic uptake via scavenger receptor B-I (SR-BI)), and indirect (via shifting cholesterol from HDL particles to apoB-containing lipoproteins) pathways (Mahdy Ali et al., 2012[67]; Siddiqi et al., 2015[103]; Pizzini et al., 2017[88]). Another potential mechanism by which *n*-3 PUFAs may exhibit their atheroprotective activities is through enhancement of intracellular catabolism of apolipoprotein B-100 lipoproteins and curbing hepatic apo-B production. This leads to stimulation of lipoprotein lipase (LPL), and increases plasma triglyceride clearance, conversion of the VLDL to LDL, and reducing the synthesis of LDL and reducing postprandial lipemia (Pizzini et al., 2017[88]; Chan et al., 2002[14], 2003[15]; Park and Harris, 2003[85]; Robinson and Stone, 2006[97]). It seems that *n*-3 PUFAs exert their effect by inhibiting sterol regulatory element-binding protein-1 (SREBP-1) mediated pathways, including the activation of Liver X Receptor (LXR), the nuclear transcription factors, Farnesoid X Receptor, Hepatocyte Nuclear Factor-4 Alpha (HNF4A), and Peroxisome Proliferator-activated Receptors (PPARs) (Pizzini et al., 2017[88]; Zuliani et al., 2009[120]). Different types of *n*-3 PUFA may elicit different activities regarding lipid metabolism; unlike DPA and DHA, which are deposited in the tissues and spared from catabolism, EPA is mostly directed towards β-oxidation (Albracht-Schulte et al., 2018[2]; Ghasemifard et al., 2015[33]). Additionally, it has been revealed that co-supplementation with EPA/ DHA or single supplementation of DHA leads to a decrease in gene expression of fatty acid synthase, LPL, hormone-sensitive lipase (HSL), and phosphoenolpyruvate carboxykinase (PEPCK) in retroperitoneal fat; however, such effects were not evident in EPA supplementation alone (Albracht-Schulte et al., 2018[2]; Raclot et al., 1997[90]) .

Although there are numerous reports of dyslipidemia in PCOS, studies investigating the effects of *n*-3 PUFA on lipid metabolism in PCOS patients are limited. However, several studies have reported an improvement in lipid regulation after *n*-3 PUFA supplementation in other metabolic disorders. In a study by Cussons et al. (2009[16]) omega-3 fatty acids led to a decrease in hepatic fat in PCOS women with hepatic steatosis. In another study, omega-3 fatty acids supplementation, in PCOS patients, significantly increased HDL-C, and decreased TC, LDL-C and TG (Mohammadi et al., 2012[74]); whilst in Rahmani et al. (2017[93]), 12 weeks co-supplementation of omega-3 fatty acids and vitamin E resulted in a significant decrease in serum triglycerides, VLDL, total, LDL- and total-/HDL-cholesterol in PCOS patients (Rahmani et al., 2017[93]). However, contrary to previous studies, Phelan et al. (2011[87]) reported no significant improvement in fasting and postprandial plasma triacylglycerol, apo-B48, total cholesterol, HDL cholesterol, and LDL cholesterol concentrations in response to LC *n*-3 PUFA supplementation for 6 weeks in PCOS sufferers (Phelan et al., 2011[87]). Notwithstanding, as only a limited number of trials have investigated *n*-3 PUFA supplementation as a conduit to improve PCOS complications, and that different types of *n*-3 PUFA may exert different activities and functions, more studies are warranted to clarify the mechanisms and effects of *n*-3 PUFA on lipid metabolism in PCOS. 

### Omega-3 and endothelial function

In addition to dyslipidemia, endothelial dysfunction, which is defined as the reduction of vasodilatory and anti-inflammatory parameters and an increase in vasoconstrictive and pro-inflammatory parameters, is considered to be another significant indicator in women with PCOS which may lead to an increased risk for atherosclerotic cardiovascular (CV) diseases (Wenner et al., 2011[112]; Abbott et al., 2002[1]). Evidence from previous studies has demonstrated that the cardioprotective effects of *n-3* PUFAs are manifest through improvements in vascular function. Several mechanisms, including decreased arterial plaque build-up (Zehr and Walker, 2018[118]; Renier et al., 1993[96]), increased anti-inflammatory properties (Zehr and Walker, 2018[118]; Calder, 2006[9]), enhanced endothelial-dependent vasodilation as measured by flow-mediated dilation (FMD) (Zehr and Walker, 2018[118]; Wiest et al., 2017[114]; Goodfellow et al., 2000[37]; Siasos et al., 2013[102]), lowering blood pressure (Zehr and Walker, 2018[118]; Ulu et al., 2014[110]; Hoshi et al., 2013[45]; Miller et al., 2014[72]), elevated antioxidant capacity (Zehr and Walker, 2018[118]; Thorlaksdottir et al., 2006[108]) and production of nitric oxide (Albracht-Schulte et al., 2018[2]; Harris et al., 1997[43]) have been suggested. In spite of the apparent dysfunction of endothelial function in PCOS women, there is a dearth of related studies investigating the potential beneficial effects of *n-3* PUFA, except for Talari et al. (2018[107]), who indicated a significant decrease in maximum levels of left carotid intima-media thickness (CIMT), mean left CIMT levels, maximum levels of right CIMT, and mean right CIMT levels after 12-week co-supplementation with Omega-3 and vitamin E (Talari et al., 2018[107]). 

### Omega-3 and hormonal factors

Hyperandrogenism is considered to be a basic characteristic of PCOS (Gunalan et al., 2018[39]; Stein et al., 2016[106]), and is often associated with excessive release of luteinizing hormone (LH) by the pituitary gland and is related with hyperinsulinemia and insulin resistance (Liepa et al., 2008[66]; Ehrmann, 2005[26]). The mechanisms by which insulin resistance impacts the hormonal system are numerous, moreover, it has been shown that insulin induces elevated androgen production and produces a hormonal milieu which results in hirsutism. The improvement in the reproductive system has been attributed to an improvement in LH concentration and LH/Follicle stimulating hormone (FSH) ratio, where the potential mechanistic action can be attributed to arachidonic acids (Nadjarzadeh et al., 2015[77]; Phelan et al., 2011[87]). Arachidonic acid can activate a steroidogenic acute regulatory protein (StAR), which is considered to be the rate-limiting tenet of the steroidogenic pathway. StAR transfers the cholesterol to the inner part of the mitochondrial membrane, which is considered the first and the most important step in the steroidogenic pathway and leads to the production of both androstenedione and testosterone. Therefore, high concentrations of arachidonic acid can result in LH-stimulated steroidogenesis, whilst current evidence suggests that the reduced availability of arachidonic acid is related to levels of omega-3 (Nadjarzadeh et al., 2015[77]; Phelan et al., 2011[87]).

Jamilian et al. (2018[48]) reported that a decreased total serum level of testosterone was evident after co-supplementation of vitamin D and omega-3 fatty acid (50,000 IU vitamin D every 2 weeks plus 2000 mg/day omega-3 fatty acid from fish oil) for 12 weeks, whilst in another study, supplementing with omega-3 fatty acids from flaxseed oil plus vitamin E for 12 weeks in PCOS women, resulted in significant reductions in serum total testosterone and free testosterone (Ebrahimi et al., 2017[25]). A similar effect of omega-3 consumption on hormonal factors in PCOS women was observed in Oner and Muderris (2013[83]); where the participants were treated with 1,500 mg of omega-3, daily, for 6 months. Resultantly, serum LH and testosterone levels decreased and sex hormone-binding globulin levels increased significantly (Oner and Muderris, 2013[83]). Contrastingly, in Nadjarzadeh et al. (2015[77]), PCOS women supplemented with 3 capsules of omega-3 (each one contained 180 mg EPA and 120 mg DHA) daily for 8 weeks, which resulted in no change in FSH. However, mean LH decreased by 1.74 mlU/ml in the omega-3 group, and the mean change in LH/FSH ratio between groups was significant; moreover, the authors reported no meaningful change in prolactin levels (Nadjarzadeh et al., 2015[77]). The aforementioned studies have predominantly explored the effect of *n*-3 PUFA supplements, Kalgaonkar et al., however, focused on dietary intake. In Kalgaonkar et al. (2011[51]), thirty-one PCOS patients consumed either walnuts or almonds containing 31 g of total fat per day for 6 weeks. Walnut consumption increased sex hormone-binding globulin, whilst almond consumption reduced free androgen index (Kalgaonkar et al., 2011[51]). 

## Conclusions

**Figure 1:**
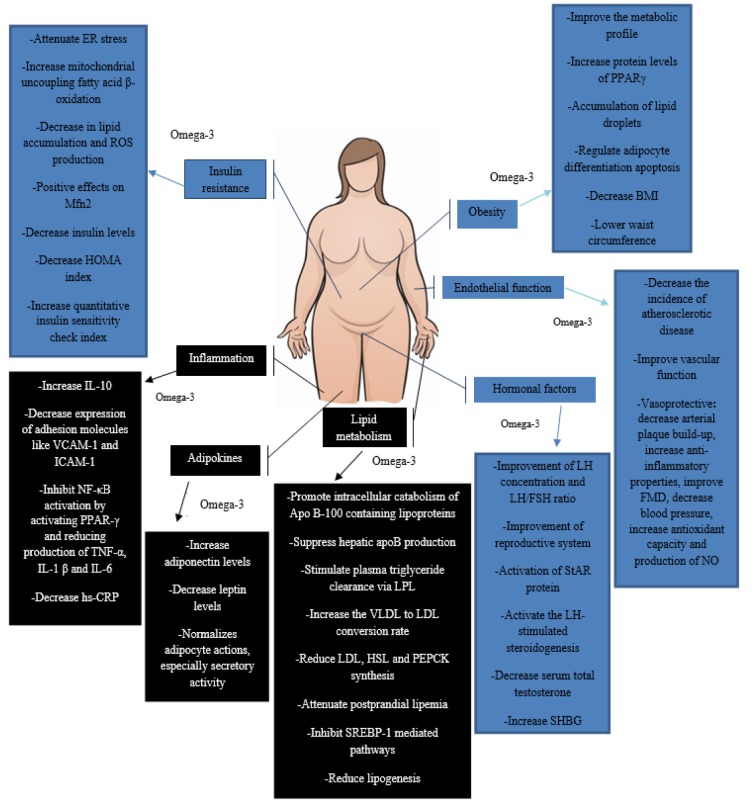
Possible mechanisms describing the effects of *n-3* PUFAs on PCOS complications. PPARγ, peroxisome proliferator-activated receptor γ; ER, endoplasmic reticulum; ROS, reactive oxygen species; Mfn2, mitochondrial fusion proteins-mitofusins 2; IL-, interleukin; TNF-α, tumour necrosis factor; VCAM-1, vascular cell adhesion molecule; ICAM-1, intercellular adhesion molecule; NF-κB, nuclear factor kappa B; hs-CRP, high-sensitivity C-reactive protein; LPL, lipoprotein lipase; VLDL, very low density lipoprotein; LDL, low density lipoprotein; HSL, hormone-sensitive lipase; PEPCK, phosphoenolpyruvate carboxykinase; SREBP-1, sterol regulatory element-binding protein-1; FMD, flow-mediated dilation; NO, nitric oxide; LH, luteinizing hormone; FSH, follicle stimulating hormone; StAR, steroidogenic acute regulatory protein; SHBG, sex hormone-binding globulin

*N*-3 PUFAs are a promising agent in relieving complications associated with PCOS. There are multiple mechanisms by which *n*-3 PUFAs may exert their beneficial effects on PCOS women, including anti-obesity, glycemic and hormonal homeostasis, anti-inflammatory, regulation of adipokine production and enhancement of endothelial function (Figure 1(Fig. 1)). Whilst studies investigating the effect of omega-3 supplements in patients with PCOS have reported an improvement in numerous complications and comorbidities, including insulin resistance, dyslipidemia, hyperandrogenism, and regulation of metabolic indicators; there is a distinct dearth of studies investigating the dietary intake of these types of fatty acids. Therefore, more studies are needed to investigate the effect of dietary fatty acids on PCOS complications. Moreover, there are, evidentially, numerous beneficial effects associated with *n*-3 PUFA consumption on PCOS complications, however, favorable effects regarding the improvement of dyslipidemia, regulation of adipokines and regulation of hormonal factors and endothelial function are limited. Therefore, given the importance of these complications in the pathogenesis of PCOS, more trials are warranted to investigate mechanisms to clarify the metabolic and hormonal effects of these agents in PCOS women. Additionally, to our knowledge, the use of *n*-3 PUFA on infertile PCOS patients has not been investigated in previous studies, and more studies are necessary to assess the role of *n*-3 PUFA in the treatment of PCOS induced infertility.

## Conflict of interest

The authors declare no conflicts of interest associated with the present manuscript.

## Figures and Tables

**Table 1:**
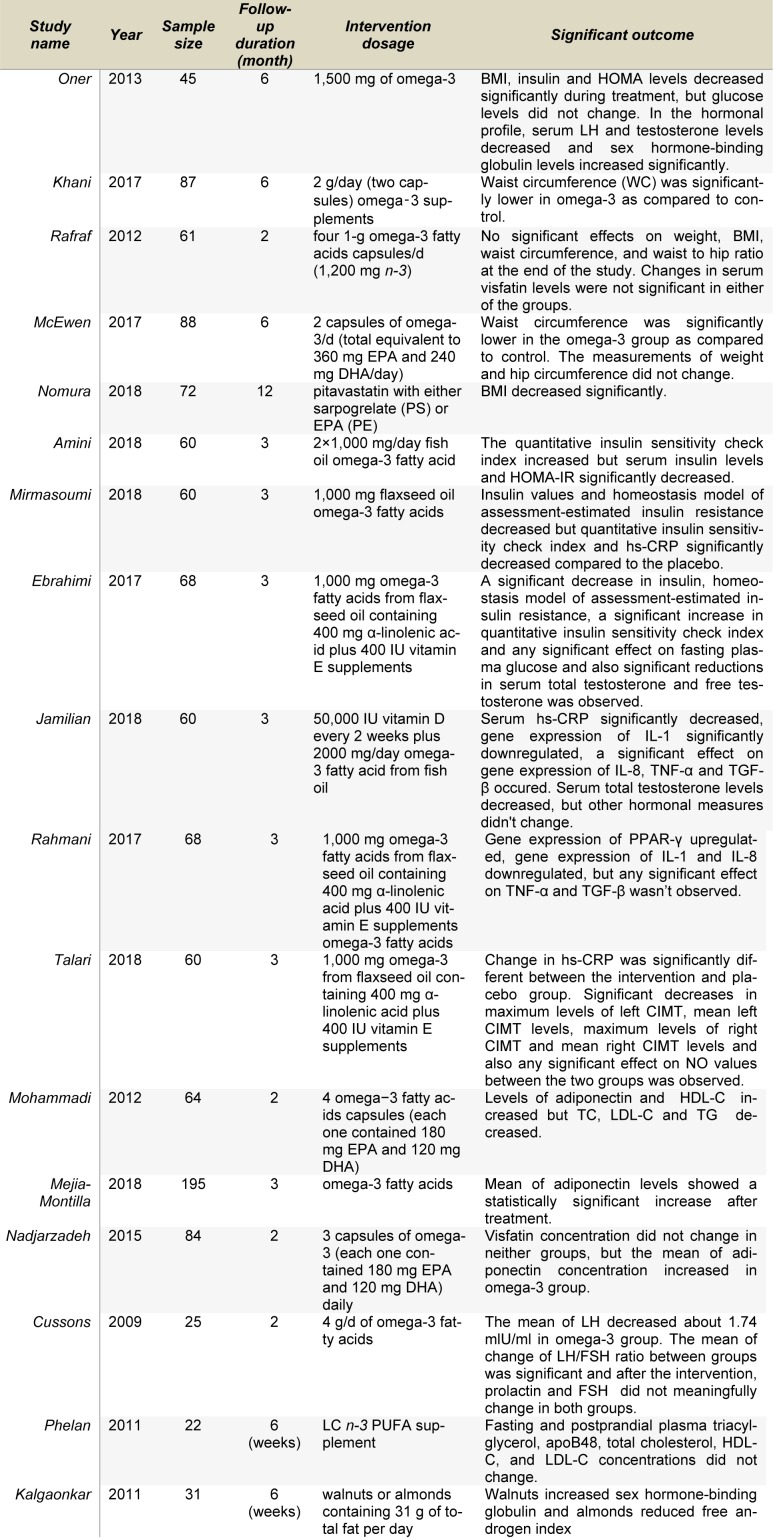
Characteristics of trials investigating effects of *n-3* PUFAs on PCOS complications
